# Strain Distribution Evaluation of Rat Tibia under Axial Compressive Load by Combining Strain Gauge Measurement and Finite Element Analysis

**DOI:** 10.1155/2019/1736763

**Published:** 2019-12-01

**Authors:** Jiazi Gao, Bei Liu, Min Zhang, He Gong, Bingzhao Gao

**Affiliations:** ^1^State Key Laboratory of Automotive Simulation and Control, Jilin University, Changchun, China; ^2^Department of Engineering Mechanics, Nanling Campus, Jilin University, Changchun, China

## Abstract

This study is aimed at providing an effective method for determining strain-load relationship and at quantifying the strain distribution within the whole tibia under axial compressive load on rats. Rat tibial models with axial compressive load were designed. Strains in three directions (0°, 45°, and 90°) at the proximal shaft of the tibia were measured by using a strain gauge rosette, which was used to calculate the maximum and minimum principal strains. Moreover, the strain at the midshaft of the tibia was measured by a single-element strain gauge. The slopes of the strain-load curves with different peak loads were calculated to assess the stability of the strain gauge measurement. Mechanical environment in the whole tibia by the axial compressive load was quantified using finite element analysis (FEA) based on microcomputed tomography images. The von Mises elastic strain distributions of the whole tibiae were evaluated. Slopes of the strain-load curves showed no significant differences among different peak loads (ANOVA; *P* > 0.05), indicating that the strain-load relationship obtained from the strain gauge measurement was reasonable and stable. The FEA results corresponded to the experimental results with an error smaller than 15% (paired Student's *t*-test, *P* > 0.05), signifying that the FEA can simulate the experiment reasonably. FEA results showed that the von Mises elastic strain was the lowest in the middle and gradually increased to both sides along the lateral direction, with the maximal von Mises elastic strain being observed on the posterior side under the distal tibiofibular synostosis. The method of strain gauge measurements and FEA used in this study can provide a feasible way to obtain the mechanical environment of the tibiae under axial compressive load on the rats and serve as a reference for further exploring the mechanical response of the bone by axial compressive load.

## 1. Introduction

Bone is a weight-bearing and mechanosensitive tissue. The adaptive responses to mechanical load in cortical and trabecular regions have been studied extensively. Animal studies can provide detailed data on bone response. As a valid method for controlled and repetitive load of the murine skeletons, the axial compressive load models were widely used.

Axial loading models in rodents included tibial loading models [1–10] and ulnar loading models [11–15]. In previous studies, these models were used to investigate the loading responses in cortical and trabecular bones [10], cortical defect repair [16], knee injury [17], and fatigue characterization [13–15]. Controlled tibial axial compressive load was confirmed to increase the cancellous bone mass and tissue density in the proximal metaphysis [3] and increase the cortical bone formation [4] and trabecular bone volume [4, 5].

The animal models have been instrumental in advancing the understanding of the response of bone to mechanical stimuli. Strain is considered one of the main factors for inducing bone tissue response to load [18]. Exact strain-load relationship under controlled load plays an important role in quantifying the local mechanical response of the bone. The strain-load relationship was determined by using strain gauge measurements, engineering beam theory, finite element analysis, or the combination of the several methods mentioned above [1, 7, 8, 19]. Strain gauge measurements of the animal can provide detailed strain data of the bone under axial compressive load. Limited by the bone shape, the bone midshaft was considered the main location for strain gauge measurement. Usually, only one single-element strain gauge was used in most studies during strain gauge measurement [1, 3, 6, 7, 18–22], whereas other studies used two or three gauges to obtain the surface bone strain under axial compressive load [4, 8]. However, strain gauge was considered highly sensitive to the exact location on the bone. The results obtained from strain gauge measurements were affected by bone shape, gauge size, and location. Thus, strain gauge measurement only is not recommended for studies addressing at identifying a reliable relationship between tissue response and local strains [18]. Finite element models based on microcomputed tomography (micro-CT) images can incorporate the microarchitecture of bone accurately [23]. Finite element analysis based on micro-CT was confirmed to be an effective way to quantify the organ- or tissue-level bone mechanical properties [3, 7, 8, 18, 19, 22, 24]. Thus, the combination of strain gauge measurement (in vivo and ex vivo) and FEA for determining the strain-load relationship and to quantify the strain distribution was recommended in several studies [7, 18, 19]. Nevertheless, for FEA, no reasonable and well-accepted model was yet available. Loading and boundary conditions have not been described carefully. For material properties, the isotropic properties of material (e.g., 10 samples with a uniform modulus of 20 GPa [3], 8 samples with 21 GPa [8], or 8 samples with 18.3 GPa [18]) were routinely applied in several studies; but the heterogeneous properties of bone tissue captured by dynamic calculation of elastic modulus based on density-modulus relationship have been proposed in other studies [7, 19, 22]. Accordingly, to provide an effective method for determining strain-load relationship, the loading and boundary conditions as well as material properties will be investigated deeply in the current study.

The mouse tibial model is usually used to investigate the strain-load relationship in other studies. However, due to the curvature of the tibiae, tissue strain distribution of the tibia under axial compression is complex. The rat models (15 samples) with axial compressive load will be used in the current study to provide an effective method to obtain the strain distribution over the whole tibiae under axial compressive load; the results will serve as reference for further exploring the mechanical response of bone to axial compressive load. Design and validation of the rat tibia loading model involved measurement of surface bone strains at the proximal shaft and the midshaft of tibia, assessment of the relationship between strains and peak loads, FEA, and the evaluation of the mechanical environment of the whole tibiae.

## 2. Materials and Methods

### 2.1. Strain Gauge Measurement of the Relationship between Load and Strain

A total of 15 5-month-old female rats were purchased (sample information is shown in Table 1). Free cage movement was allowed with standard rat chow and tap water before experiments. All the procedures were approved by the Ethics Committee of The First Hospital of Jilin University (No. 2018-238).

All rats were euthanized, and the intact right hindlimbs were harvested and immediately prepared for strain gauge measurements. After the knee was fixed, the tibial length was measured by using a Vernier caliper. An incision was made on the lateral aspect of the tibia, and the skin and muscle attachments were removed. After gently removing the periosteum, the bone surface was exposed and then degreased by acetone.

Strains in three directions (0°, 45°, and 90°) at the proximal shaft of the tibia were measured by using a strain gauge rosette. The maximum principal strains *ε*_max_ and minimum principal strains *ε*_min_ were calculated based on

(1) $$\begin{array}{c} \varepsilon_{\max}=\frac{\varepsilon_{0}+\varepsilon_{90}}{2}+\frac{1}{2}\sqrt{{\left(\varepsilon_{0}-\varepsilon_{90}\right)}^{2}+{\left(2\varepsilon_{45}-\varepsilon_{0}-\varepsilon_{90}\right)}^{2}}, \end{array}$$

(2) $$\begin{array}{c} \varepsilon_{\min}=\frac{\varepsilon_{0}+\varepsilon_{90}}{2}-\frac{1}{2}\sqrt{{\left(\varepsilon_{0}-\varepsilon_{90}\right)}^{2}+{\left(2\varepsilon_{45}-\varepsilon_{0}-\varepsilon_{90}\right)}^{2}}, \end{array}$$

where *ε*_0_, *ε*_45_, and *ε*_90_ were the strain in 0°, 45°, and 90° directions, respectively.

**Figure 1:**
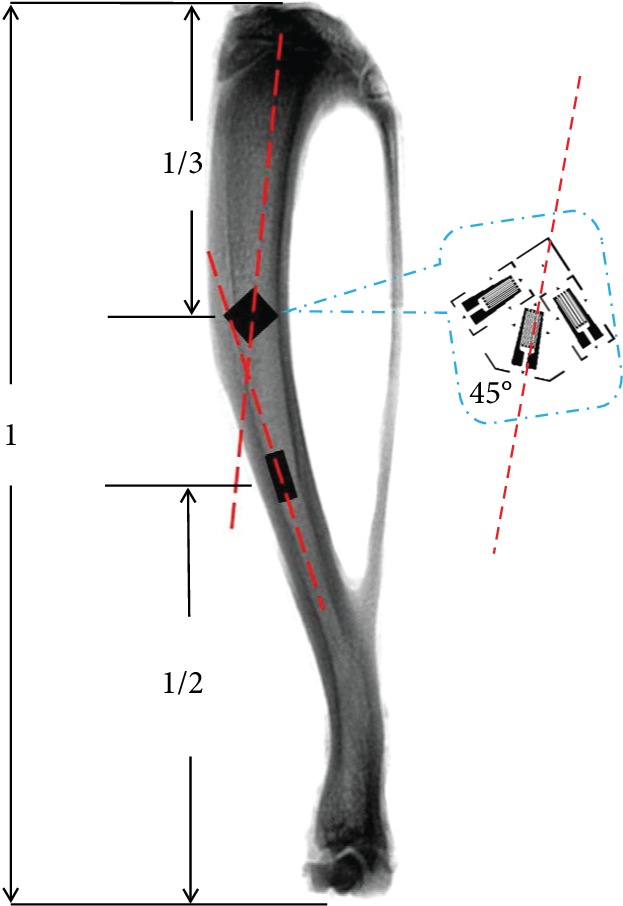
Locations of strain gauges (red line: long axis).

Moreover, the strain at the midshaft of the tibia was measured by using a single-element strain gauge. All gauges were waterproofed for 12 h before experiment, and gauges with resistance values outside the range of 120 ± 0.5 *Ω* were excluded. The single-element strain gauge was then attached to the surface of the tibial midshaft (1/2 tibial length) aligned with the long axis of the bone, and the strain gauge rosette was attached to the surface at 1/3 tibial length from the tibial plateau, with the 45° gauge aligning with the long axis of the bone (Figure 1).

**Figure 2:**
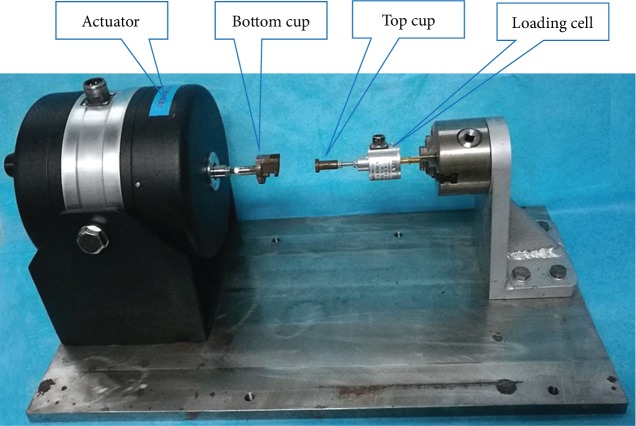
Custom-made dynamic loading device.

**Figure 3:**
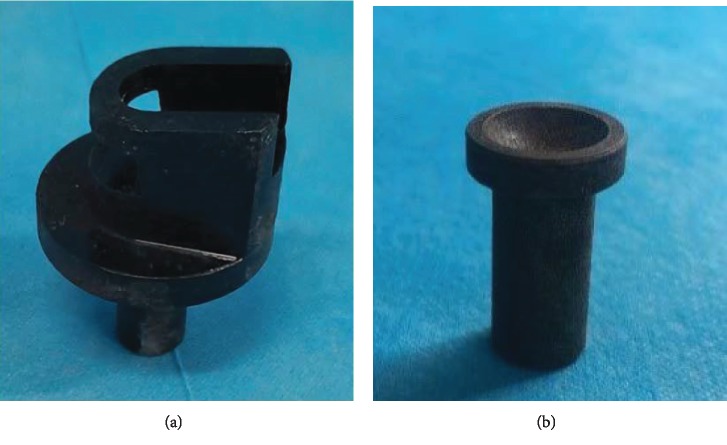
Fixed cups of rat tibia in the custom-made dynamic loading device: (a) the bottom cup and (b) the top cup.

Cyclic dynamic axial compressive load was applied through a custom-made dynamic loading device (Figure 2). Meanwhile, a couple of custom-made cups were used in the study [4, 5, 16] (Figure 3). In other words, the top cup presents a concavity to hold the flexed knee, and the bottom cup was designed based on the morphology of rat ankle at an approximately 45° slope to hold the ankle. The cups were aligned horizontally, with the upper cup being attached to the tension-compression loading cell and the lower cup being attached to the actuator (Figure 2). The strain gauges and the loading cell were connected to a dynamic signal analysis system to collect the strains and loads. The sampling frequency of dynamic signal analysis system was set as 500 Hz.

**Figure 4:**
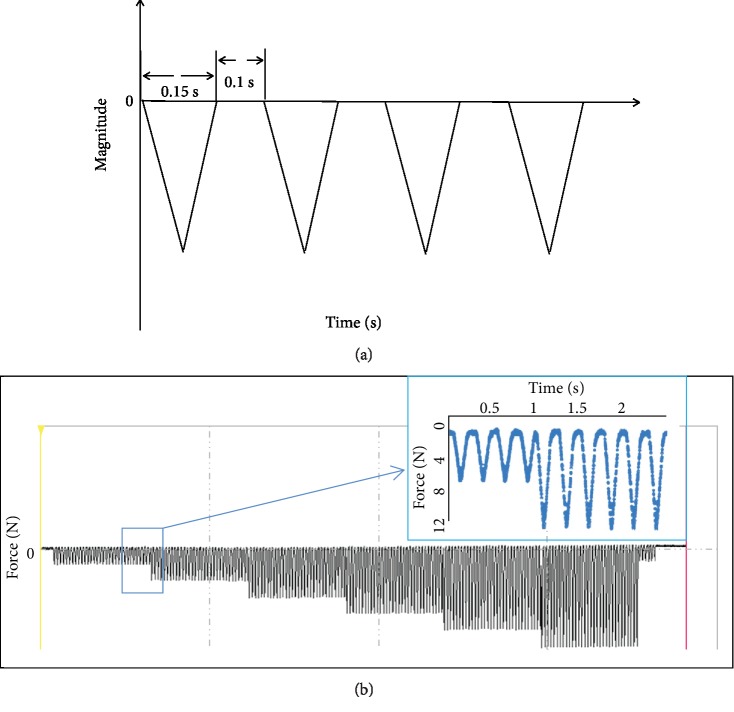
Cyclic dynamic compressive load: (a) the input waveform and (b) the process of 6-step loading regime; blue arrow: the loading waveform between the first and the second loading steps.

A 4 Hz triangle waveform including 0.15 s of symmetric loading/unloading and 0.1 s rest in a cycle was applied to each tibia [19] (Figure 4(a)). To maintain the initial position and avoid strain drift of the dynamic signal analysis system, a preload of −10 N was applied for 10 min before the axial compressive load. Peak loads of −20, −30, and −40 N were performed. To avoid the impact during loading, a 6-step loading regime was used in the study, that is, the peak load was increased from −10 N with 16.67% increment to the targeted peak load, with each step being maintained for 8 s (a total of 48 s with 192 cycles, Figure 4(b)). The maximum principal strains, the minimum principal strains, and the midshaft strains at the last step (strains under the targeted peak load) were calculated.

### 2.2. FEA and Strain Distribution Analysis

Finite element models based on micro-CT scanning were built. The mechanical parameters in the whole tibiae under axial compressive loads were quantified by static linear elastic FEA.

#### 2.2.1. Finite Element Models Based on Micro-CT

**Figure 5:**
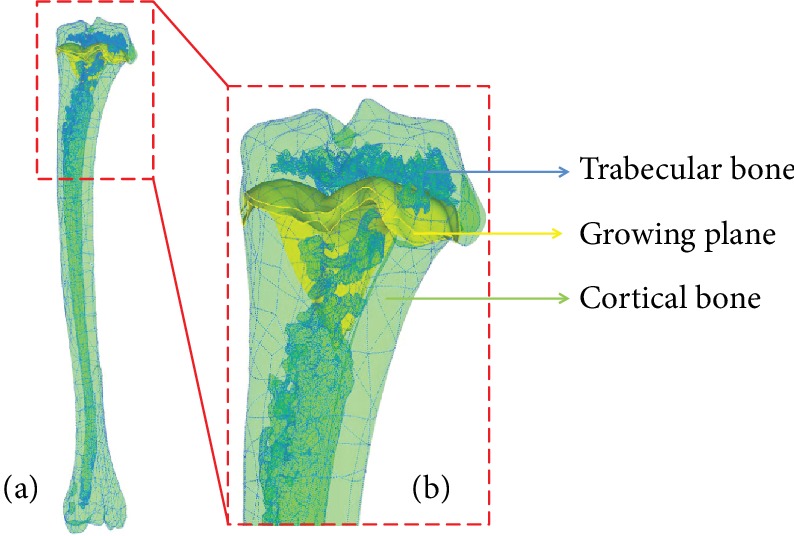
Geometry of the tibia: (a) the whole tibia model and (b) the proximal tibia.

After strain gauge measurement, the samples were moved from the experimental cups to a couple of acrylonitrile butadiene styrene (ABS) cups. The ABS cups were 3D printed by a 3D-printing device in accordance with the cups used in the experimental study. The positions of the hindlimbs in the ABS and experimental cups were certified consistently. In other words, the relative positions of the hindlimbs in the ABS and experimental cups were consistent. The ABS cups were then fixed by two ABS screws. Before micro-CT scanning, the wires of gauges were cut off, followed by complete polishing of metallic explosion, and the gauge bases were maintained to confirm the gauge locations in micro-CT images, which can provide an accurate region for strains compared in FEA. Micro-CT scanning was performed by a micro-CT system operated at 79 kV and 125 *μ*A with Al 1.0 mm filter. The spatial resolution for specimen scanning was set to 18 *μ*m. The 3D reconstruction of the micro-CT images was performed by using Materialise Mimics® to acquire accurate bone geometry. The medulla of the tibia and holes in the trabecular bone were reserved (Figure 5; the fibula was omitted in order to avoid affecting the indications of the tibia).

#### 2.2.2. Meshing and Element Type

The geometric models were converted to finite element in Hypermesh®. That is, 3D solid models built in Materialise Mimics® were exported to Hypermesh® and subdivided into discrete elements. The models were meshed automatically and average edge length was 150 *μ*m.

Since the experimental strains were well correlated with the FEA results using the second-order tetrahedral finite elements [25], the tibial models were meshed using the 10-node quadratic element SOLID187 in Hypermesh® [26, 27]. All tibiae tested by strain gauge measurement were simulated in FEA, that is, a total of 15 finite element models based on micro-CT scanning built. The number of tetrahedral elements ranged from 856,074 to 1,203,473, the number of nodes ranged from 1,250,322 to 1,756,902, and the minimum element sizes ranged from 74 *μ*m to 119 *μ*m.

#### 2.2.3. Boundary and Loading Conditions

**Figure 6:**
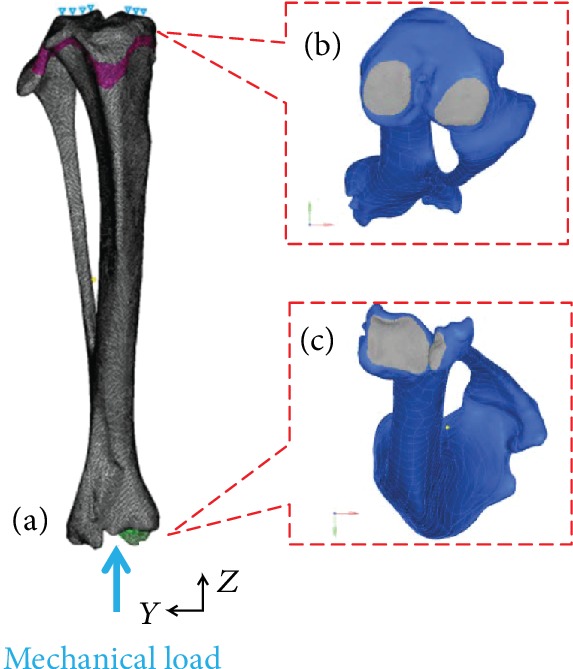
Typical finite element model of the rat tibia: (a) boundary and loading conditions of the finite element model; (b) the fixed bone surface on the tibial plateau; (c) the selected surface for mechanical load on the distal tibia.

Based on the positions of the fixed cups in the micro-CT images, the mechanical load direction was defined directly. The *z*-axis corresponded to the experimental loading axis with the *y*-axis paralleling to the sagittal plane. Geometrical models including the joint contact surfaces (proximal and distal ends of the tibia) were obtained based on the micro-CT images in Materialise Mimics®. These models were then exported to Hypermesh® and used as a reference for boundary conditions. Nodes on the boundaries were chosen manually to match the experimental conditions. Mechanical load was applied through a contact pressure surface selected on the distal end, as described by micro-CT images (Figure 6(c)). On the knee side, the tibiofemoral contact nodes were all fixed on X and Z directions (Figure 6(b)).

#### 2.2.4. Material Properties

Material properties were defined as described in Razi et al. [22]. In brief, 20 materials were used in the study, and the elastic modulus values were set based on the ash mineral density:

(3) $$\begin{array}{c} E=k{\left(\rho_{\text{ash}}\right)}^{1.5}, \end{array}$$

where *E* is Young's modulus; *ρ*_ash_ represents the ash mineral density, and *k* is a constant. Considering a linear relationship between ash density and linear attenuation coefficient, equation (3) becomes

(4) $$\begin{array}{c} \frac{E_{{\text{ROI}}_{a}}}{E_{{\text{ROI}}_{b}}}={\left(\frac{{\hat{\mu }}_{a}}{{\hat{\mu }}_{b}}\right)}^{1.5}, \end{array}$$

where *μ* is the linear attenuation coefficient; ROI is the region of interest; and ${\hat{\mu }}_{a}$ and ${\hat{\mu }}_{b}$ are the average *μ* in ROI_*a*_ and ROI_*b*_, respectively.

Based on the above equations, the maximum Young's modulus (*E*) should reasonably represent the upper boundary of the elastic modulus for rat tibiae. To obtain the reasonable elastic modulus for rat tibia, a nanoindentation test was designed. Five 5-month female rats were selected, and the longitudinal cortical bones of tibiae (five samples) were cut. Nanoindentation test and determined parameters were used as described previously [28–30]. Specifically, longitudinal cortical bones with a thickness of 2 mm were cut from tibial shafts. After cutting, the specimens were dehydrated in a series of alcohol baths (70, 80, 90, and 100% for 48 h each period) and then embedded in epoxy resin. All the embedded samples were polished using abrasive silicon carbide papers with decreasing particle sizes (600, 800, and 1200 grit) for the nanoindentation test. Nanoindentation tests were performed using the Nano Indenter G200. The indentation modulus (GPa) was calculated using Oliver and Pharr's method and collected by the nanoindentation test software. The results showed the indentation modulus of longitudinal cortical bone of 18 GPa, which was assumed to represent the upper boundary of elastic modulus for rat tibiae in this study.

**Figure 7:**
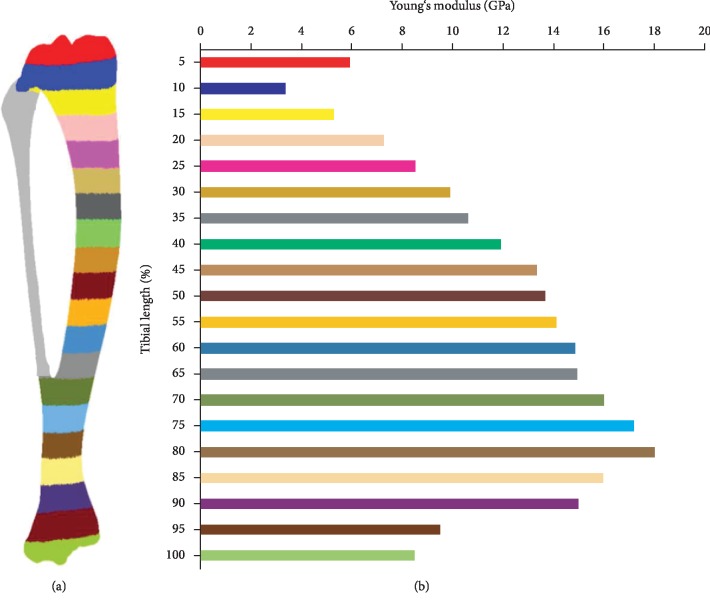
Typical material properties of the tibia model: (a) the tibia model and (b) material property distribution of the whole tibia. The meshes were hidden in order to avoid affecting the indications of the material properties.

The whole tibia was equally divided into 20 regions along the tibial axis. The ash mineral density of each region was calculated in Materialise Mimics®, and different Young's moduli were derived based on the above equations. Young's moduli were then assigned to the corresponding regions manually. Material property of the fibula was defined separately, and Young's modulus was set as 5 GPa [22] (Figure 7). A Poisson's ratio of 0.3 was assigned to all the models [8].

Given the linear relationship between loads and bone strains under axial compressive load, a −40 N load was applied to the finite element models. The linear elastic FEA was performed in ANSYS®.

#### 2.2.5. Gauge Node Set and Local Coordinate System

**Figure 8:**
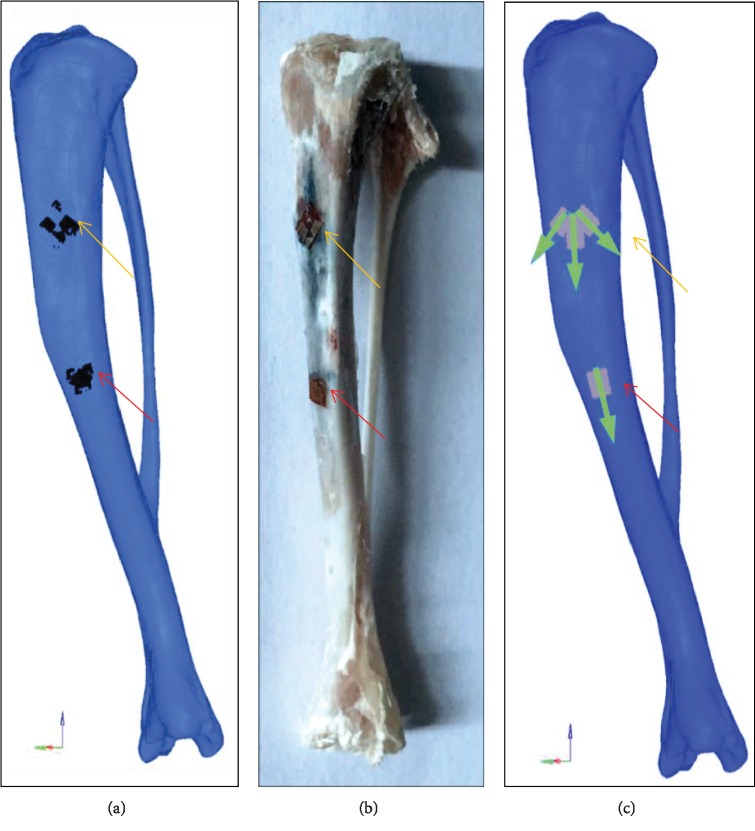
Regions of strain gauges and local coordinate system: (a) regions of strain gauges scanned by micro-CT; (b) regions of strain gauges on the samples; (c) node sets of strain gauges and local coordinate systems. Red arrow: single-element strain gauge; yellow arrow: strain gauge rosette; green arrow: *z*-axis of the local coordinate system.

Node sets of the strain gauges and local coordinate system were established to obtain accurate strains in FEA (Figure 8). Specifically, the 3D model including the location information of strain gauges was imported to Hypermesh® (Figure 8(a)), wherein the 3D model was coincided with the tibia model. Thus, the locations of strain gauges can be observed, and the gauge node sets can be selected manually on the surfaces of the tibia models. Moreover, the local coordinate systems were defined for strain analysis, where the *z*-axis was oriented along the measurement axis and the *y*-axis was parallel to the measurement plate (Figure 8(c)). The strain at the gauge site was calculated by averaging the nodal strains over the gauge regions.

### 2.3. Statistical Analysis

The relationship of mechanical load and strain was defined by using the slope of the strain-load curve. The average and standard deviation of the slope were then compared between the results of the experiment and FEA. One-way analysis of variance (ANOVA) followed by least significant difference (LSD) test was used to compare the means of slopes under different peak loads. Analyses of differences between the experimental and computational strains were performed using paired Student's *t*-test. The linear correlation between the experimental and computational results and the loads and strains under axial compressive loads were assessed by using Pearson's squared correlation coefficient (*R*^2^). The statistical significance was set at *P* < 0.05.

## 3. Results

### 3.1. Relationship of Strains and Loads Obtained by Strain Gauge Measurement

**Figure 9:**
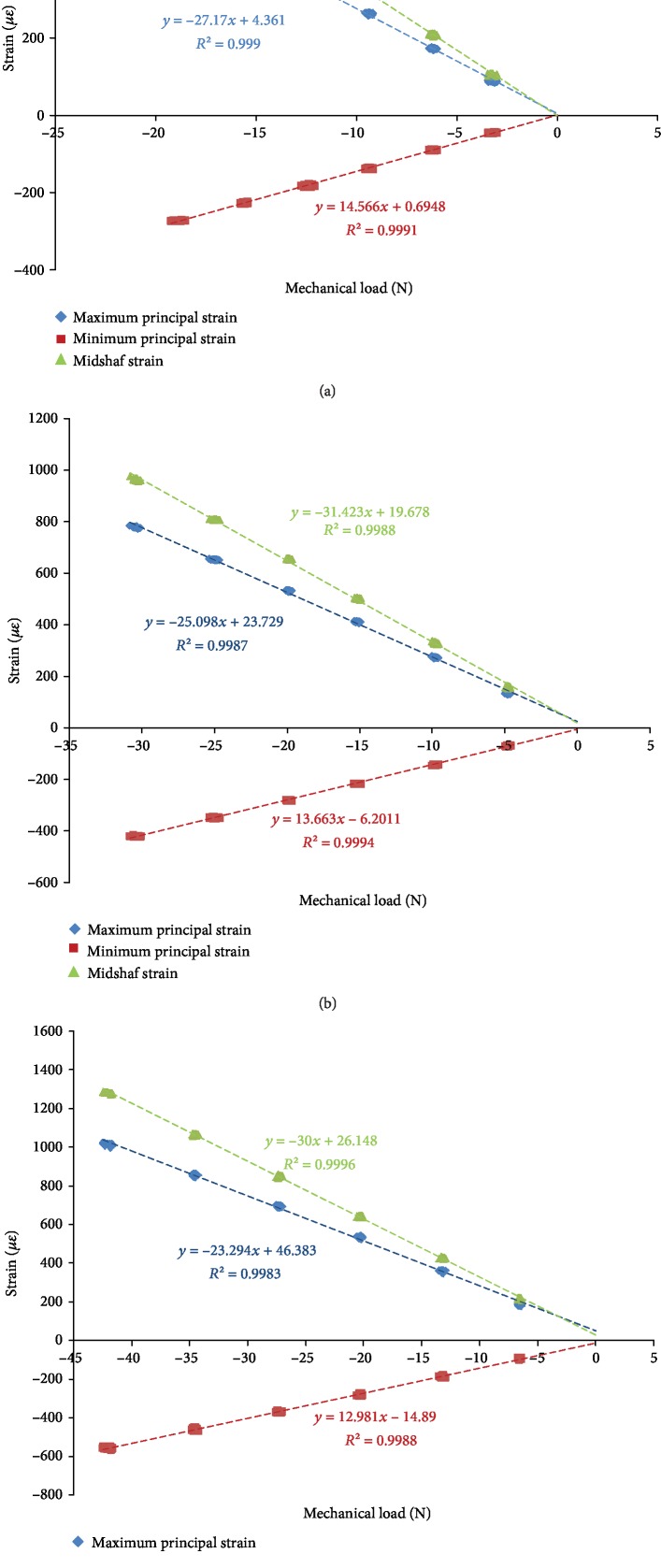
Typical strain-load curves under different peak axial compressive loads: (a) −20 N peak load; (b) −30 N peak load; (c) −40 N peak load.

Figure 9 shows the typical strain-load curves under different peak axial compressive loads. The results showed that the strains (including the maximum principal strains, minimum principal strains, and the midshaft strains) and loads satisfied the linear relation.

Table 2 shows the slopes of the strain-load curves obtained via experimental measurement. ANOVA showed no significant difference in the slopes among different peak loads (*P* > 0.05), thus indicating that the strain-load relationship obtained from the tibia models was reasonable and stable.

### 3.2. FEA Model Validation

**Figure 10:**
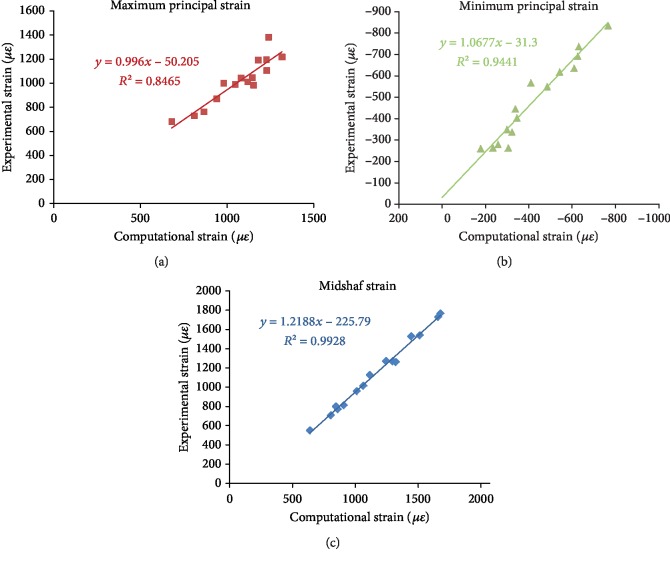
Linear regressions of the computational and experimental strains: (a) maximum principal strain; (b) minimum principal strain; (c) midshaft strain.

The relationships between the experimental and computational results (i.e., the maximum principal strain, the minimum principal strain, and the midshaft strain) were linear with *R*^2^ > 0.8 (*P* < 0.05). The computational strains correlated closely to the experimental values in all tibial regions (*P* < 0.05), which shows a strong correlation between the experimental and computational results (Figure 10).  Table 3 shows the strains (including the maximum principal strain, minimum principal strain, and midshaft strain) obtained by strain gauge measurement and FEA under the mechanical load of −40 N. Paired Student's *t*-test showed no significant differences between the experimental results and FEA results (*P* > 0.05). The FEA results corresponded to the strain gauge measurements with an error smaller than 15%, indicating that the FEA can reasonably simulate the experimental results and the strain distribution of other regions throughout the tibia can be extrapolated by using the FEA models.

### 3.3. von Mises Elastic Strain Distribution of the Whole Tibia

**Figure 11:**
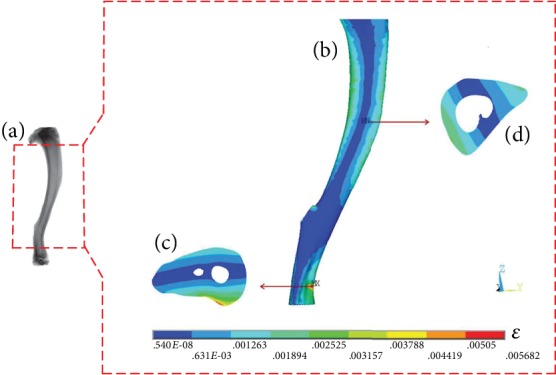
Typical von Mises elastic strain distribution of the tibial midshaft: (a) image of the tibia scanned by micro-CT; (b) von Mises elastic strain distribution of the tibial midshaft; (c) the cross section with the maximal strain; (d) the cross section with the minimal strain; red dotted box: the tibial midshaft selected for analyzing the strain distribution.

The von Mises elastic strain distribution of the whole tibia was evaluated (Figure 11; the fibula was omitted in order to avoid affecting the indications of the tibia). To avoid the influence of stress concentration on the irregular epiphysis, 80% of the tibial length (measured from the most proximal end to the most distal end of the tibia) was selected to analyze the strain distribution.

For the tibial midshaft, the von Mises elastic strain was the lowest in the middle and gradually increased to both sides along the lateral direction with the maximal von Mises elastic strain being observed on the posterior side under the distal tibiofibular synostosis. Figure 11 shows the typical von Mises elastic strain distribution of the whole tibial midshaft and the strain on the cross section with the maximal or minimal strain region.

## 4. Discussion

The current study combined strain gauge measurement and FEA method to obtain the relationship between loads and tibial strains. The goal was to provide a loading model to observe bone adaptation to mechanical environment and to understand the cellular and biological pathways of the cell response under mechanical stimulation.

Mouse models (experimental and computational models) are the most frequent models used in the previous studies. However, mouse tibiae were too small to place a gauge to confirm the numerical results [7]. Accordingly, rat tibiae used in the study can overcome the insufficient size for gauge attachment during strain gauge measurements. Moreover, rats can provide enough samples for histology or histomorphometry analysis in the future studies, which makes the rat models to be used more widely than mouse models.

The finite element models built from micro-CT images could provide the microarchitecture of bone accurately (include trabeculae). Finite element models which included trabeculae could better describe bone architecture and made the obtained computational results more closely to reality, which were widely used for characterizing the mechanical environment in the whole tibia [3, 7, 8, 18]. Precise correlation between the applied external mechanical stimulus and biological response can be obtained using the combined (experimental/numerical) approach [7]. The method used in this study can improve the validation of the FEA models by comparing not only the strains measured by single-element strain gauges but also the maximum and minimum principal strains measured by strain gauge rosettes.

In the current study, material property arrangements of the finite element models based on micro-CT were investigated strictly before all samples were analyzed. Heterogeneous and homogeneous bone tissue material properties were used to evaluate the whole bone strain distribution [3, 7, 8, 18, 19, 22, 31]. Thus, based on previous studies, several methods of assigning bone tissue material properties with the same loading and boundary conditions were tested.

Five samples were randomly selected from the 15 tibiae. Then, the finite element models in Hypermesh® were subdivided into four parts including cortical bone, growing plane, trabecular bone, and fibula. The material properties of different parts were defined in Table 4. Poisson's ratio of 0.3 was assigned.

The FEA results with different material properties were compared. The node set strains were calculated, and the differences between FEA strains and experimental strains were compared.

**Figure 12:**
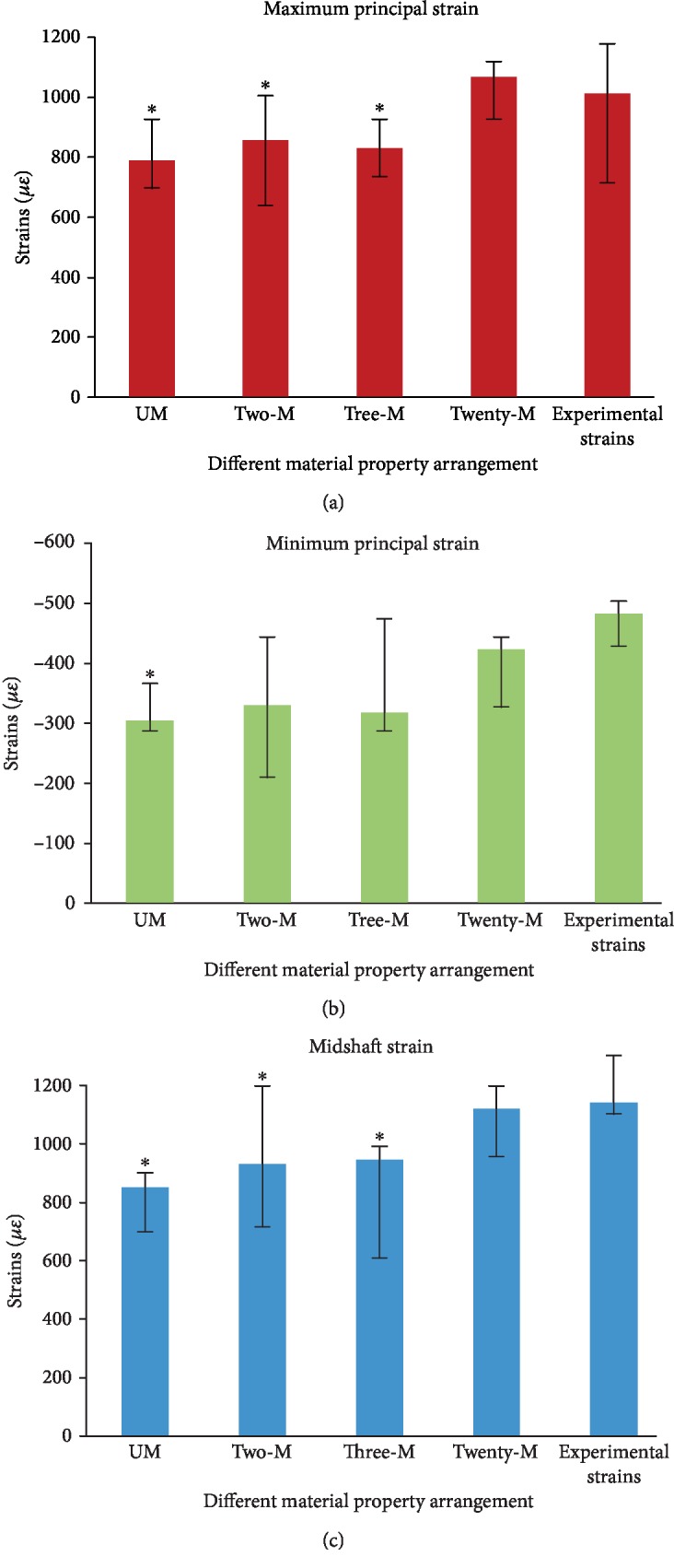
Comparisons of FEA results from different material properties of the tibiae: (a) maximum principal strain; (b) minimum principal strain; (c) midshaft strain; UM~Twenty-M: strains of FEA under different material property distribution. ^∗^Significantly different from the experimental results from ANOVA followed by LSD; *P* < 0.05.

Strains calculated by using FEA showed that significant differences were observed between UM, Two-M, Three-M, and experimental strains (ANOVA followed by LSD, *P* < 0.05, Figure 12). No significant difference between Twenty-M and experimental strains was found (ANOVA followed by LSD, *P* > 0.05), thus indicating that this method of arrangement for bone tissue material property provided the best match between the experimental and computational results. Therefore, in the study, Twenty-M (i.e., 20 materials based on the ash mineral density) was used in the subsequent FEA. In addition, the maximum tissue modulus used for material property arrangement was obtained by using the nanoindentation test. Instead of macro mechanical test or beam theory [32], the nanoindentation test can directly obtain the tissue-mechanical property, which may improve the accuracy of the material properties and the FEA reliability.

Because of the differences in strain gauge locations and the alignment errors of strain gauges, large intraindividual variability was found during strain gauge measurement [3, 18], which was also observed in the current study. Though the tibial length, locations, and long axis directions of the strain gauges, as well as the quality of strain gauges, were controlled strictly in the study, the experimental results still showed large SD values. Fortunately, the strain-load relationship obtained from strain gauge measurement was stable, which may provide an effective method for quantifying the strain distribution within the whole tibia under axial compressive load on rats.

In consideration of the larger size of bone tissue of rat than mouse, there are some differences on finite element modeling. A simplified tibial model was developed in the current study, which was convenient for meshing and cost less computational time. Although it included certain simplification, it contains most of the information about the bony tissue (including cortical bone, trabecular bone, and growing plane). The results of FEA confirmed that the computational strains matched well with the experimental results, so the simplification of model was effective.

In order to verify the accuracy of the mesh density used in the current study, a mesh sensitivity study was performed. A typical finite element model was meshed using three different element sizes (the average edge length was 75 *μ*m, 100 *μ*m, and 150 *μ*m, respectively). The total strain energy and displacement were served as the convergence criteria with the tolerance level less than 5%. Results showed that the changes were less than 1% for displacement and less than 5% for total strain energy with the edge length from 75 *μ*m to 150 *μ*m. So the element size of 150 *μ*m is considered as a reliable mesh size.

For boundary conditions, all directions of the constrained terminal were fixed in most studies. However, the constrained terminal used in our study was the knee side and the fixed cup contacted directly with the distal femur. Given the relative location of the femur trochlear and tibial patella, a minimal slide on the Y direction can be observed. For this reason, the Y direction was not fixed in the study, whereas both X and Z directions were fixed.

Strong correlations between the experimental and computational strains were observed at the gauge locations, to indicate that the mechanical environment of the whole tibiae can be extrapolated effectively by using FEA.

Studies demonstrated that the measured strains were heavily dependent on the strain gauge location, that is, even slight difference in gauge location between specimens would induce obvious variation in the measured strains [33]. To avoid this problem, the samples were selected strictly in this study. Rats with identical weight and tibial length were selected, and the gauge locations were determined by using tibial length (the single-element strain gauge was attached to the surface at 1/2 tibial length, and the strain gauge rosette was attached to the surface at 1/3 tibial length). In addition, the positions of the measured strains in FEA were defined by the micro-CT images, which can improve the rationality of the comparisons between the experimental and computational results.

Octahedral shear strain can analyze the correlation between the strain and local biological response [7], but von Mises elastic strain is often used to quantify the mechanical behavior of the whole bone and assess the bone failure at the tissue level in FEA [33, 34]. Thus, in this study, von Mises elastic strain distributions of the whole tibiae were calculated, and the mechanical environments under axial compressive load were evaluated.

The maximal von Mises elastic strain was observed on the posterior side under the distal tibiofibular synostosis. Given that a maximal mechanical stimulus will generate maximal response of the tissue [7], the bone adaptation and histological variation should be quantified as a matter of priority in these areas. Axial compressive load models were considered to be a valid method for controlled and repetitive load of the murine skeletons. However, there were limited axial compressive load models of rat. Because of the natural curvature of the tibia, compression and bending were generated along the tibia under axial compressive load. The usage of two types of gauges (strain gauge rosette for assessment of maximum and minimum principal strains, single-element strain gauge for assessment of midshaft strain) could collect the strains of the tibial surface more accurately and provide a more powerful method for strain-load relationship assessment. Rat models show obvious advantages on bone-related studies, e.g., they can provide more bone samples from a single individual than mice. Adequate samples allow deeper investigation, i.e., multiscale morphologies and mechanical properties. These may be beneficial for exploring the mechanism of bone-related disease. In general, the rat studies show obviously irreplaceable advantage over the mouse studies. Rats are recommended and expected to be studied widely in future studies.

## 5. Limitation

First, limited by the gauge size and bone morphology, only two regions were selected to be measured. Fortunately, the relationship of strain-load showed obvious linear relationship, and the computational strains matched well with the experimental results. Thus, the method used in this study can reflect the real mechanical environment. Second, cyclic dynamic compressive load was applied during strain gauge measurement, whereas static linear elastic FEA was performed in the study. This limitation has been pointed in a previous study [19], but we have not overcome the difficulty in the dynamic analysis in FEA. To understand the adaptive response of bone to mechanical stimulus, dynamic features should be investigated in the future studies. Third, ex vivo samples were used in this study instead of in vivo samples. Though the hindlimbs were collected with all the skins and muscles preserved and the bones were tested as soon as the samples were separated, there still may be influence to the bone responses during the loading periods. Thus, to gain further understanding in the bone response to mechanical stimulus, in vivo samples should be used. Fourth, no more accurate material models (>20 material properties) were built in the current study. Accordingly, it has not been assessed whether the strain-load relationship may change when more accurate material models are implemented, which needs further investigation.

## 6. Conclusion

This study combined the strain gauge measurement and FEA to obtain the strain distribution of the whole rat tibia under axial compressive load. The loading and boundary conditions and the material properties were investigated in detail. The method of strain gauge measurements and FEA used in this study can provide a feasible way to obtain the mechanical environment of the tibia under axial compressive load on the rats. The results of this study conclude that strain is directly related to mechanical stimulus. The obtained position with the highest strain could contribute to the study of cellular and biological pathways of the cell response to mechanical stimulation.

## Figures and Tables

**Table 1:** Sample information (mean ± SD).

Numbers	Body weight (g)	Tibial length (mm)
15	311.86 ± 9.76	50.71 ± 0.69

**Table 2:** The slopes of the strain-load curves obtained by strain gauge measurement (mean ± SD).

	−20 N (*με*/N)	−30 N (*με*/N)	−40 N (*με*/N)
Maximum principal strain	26.64 ± 4.86	25.49 ± 4.73	23.77 ± 5.24
Minimum principal strain	−12.41 ± 5.12	−12.39 ± 4.83	−11.36 ± 4.61
Midshaft strain	29.94 ± 9.86	28.31 ± 9.73	27.26 ± 9.39

**Table 3:** Comparison of the strains obtained by strain gauge measurement and FEA (mean ± SD).

	Experimental result (*με*)	FEA result (*με*)	Error (%)	*P* value
Maximum principal strain	1011.97 ± 194.54	1066.42 ± 179.71	5.44	0.984
Minimum principal strain	−482.13 ± 192.10	−422.23 ± 174.82	12.45	0.546
Midshaft strain	1140.19 ± 381.91	1120.76 ± 312.22	1.75	0.394

**Table 4:** Different material property distribution used in the study (GPa).

Group	Abbreviation	Cortical bone	Growing plane	Trabecular bone	Fibula
Uniform material property distribution	UM	15	15	15	15
Two-material property distribution	Two-M	15	4	15	15
Three-material property distribution	Three-M	15	4	0.5	15
Twenty-material property distribution	Twenty-M	20 regions based on the ash mineral density			5

## Data Availability

The data used to support the findings of this study are included within the article.
